# The role of stress in implementation of Chronic Fatigue Syndrome. Integrative approach to correction of postural, cognitive disorders and cerebral metabolism

**DOI:** 10.1186/1878-5085-5-S1-A149

**Published:** 2014-02-11

**Authors:** Olga Safonicheva, Marina Safonicheva, Andrei S Glotov

**Affiliations:** 1 First Moscow State Medical University by I.M. Sechenov, Moscow, Russia

## 

Development the personalized programs of rehabilitation for inhabitants of industrial cities with Chronic Fatigue Syndrome (CFS) and stress-induced diseases is actual up-to-date problem.

## Materials and methods

85 patients (38 men and 47 women, age of 48±6 years) with combined myofascial pain in the cervical part of the vertebral column and chronic fatigue syndrome were examined. Clinical neurological examination was conducted to identify the role of myotonic neck “tunnel- syndromes” and biomechanically significant biomarkers in the cervical spine for affect the cerebral blood flow. EEG- examination was carried out to assess the state of brain activity, the definition of cortical areas responsive to the effects of stress factors and hemispheric interactions, as correlates of memory. The NEC-method of visualization used to study the biochemical markers – the level of cerebral metabolism and adaptation possibilities. 15 patients used genetic test for indication genes of metabolism (genes of phase I and phase II).

## Discussion

Main complains in patients were: headache, fatigue, memory, sleep problems. Disorders in the cognitive, personal, emotional status had all patients. Neuro-vertebral examination had revealed biomechanical markers-disturbances of muscular coordination, muscular hypertension, rigidity in the shoulder girdle, syndrome of upper aperture, kyphosis, postural displacement and the lymphodinamic disturbances – edematosity of the axillary and subclavicular areas. EEG and NEC analysis revealed signs of functional inter-hemispheric asymmetry of bioelectrical activity in 80% of patients and different levels of cerebral acidosis as biomarker for brain hypoxia. Genetic tests revealed decreasing genes activity of phase II detoxification in 7 patients. Clinical and instrumental observations allow us to offer some statements. New mechanisms of non-specific base of CFS are: stress – short term or long term muscle spasm and lack of oxygen increase the density of muscles and lead to 3-level “tunnels” intervertebral (compression of nerves), intramuscular (compression of blood vessels) and intrafascial (compression of lymphatic and nodes), Retrograde lymph flow and intoxication of the intersticial matrix are suppose to be the grain for CFS and specific pathologidcal processes. The personalized integrative schemes of rehabilitation were worked out for patients with different postural, neurovascular and lymphodinamic disturbances for correction of muscle-tonic syndrome in the neck, cranio-vertebral region, for improving the cerebral metabolism and liquorodynamics. Methods of intermitted hypoxia therapy were used in the case of cerebral acidosis; adoptive gymnastics was used for patients with postural problems; nutrigenomic diet was recommended to patients for increasing phase II of detoxification.

## Conclusions

Comprehensive personalized rehabilitation of the patients with CFS improved their clinical and emotional background, memory and cognitive functions. EEG and NEC examination marked the trends in recovery of inter-hemispheric connections, normalization of the bioelectrical activity in the brain and improvement of cerebral metabolism.

